# The molecular mechanisms of signal pathway activating effect of E2F-1/NF-κB/GSK-3β on cognitive dysfunction of Alzheimer rats

**DOI:** 10.1080/21655979.2021.1989261

**Published:** 2021-11-27

**Authors:** Mayila Tuerxun, Ajar Muhda, Lixin Yin

**Affiliations:** Department of Neurology, The First Affiliated Hospital of Xinjiang Medical University, Urumqi, Xinjiang Uygur Autonomous Region, China

**Keywords:** Alzheimer’s disease, cognitive learning ability, Tau protein phosphorylation, E2F-1/NF-κB/GSK-3β signaling pathway

## Abstract

Alzheimer disease (AD) seriously harms human health and its onset is insidious. Therefore, it is of great significance to find out the pathogenesis of AD disease for improving the prevention and treatment effect of the disease. The study drew attention to the influence of E2F-1/NF-κB/GSK-3β signaling pathway on cognitive dysfunction of Alzheimer rats. 60 specific pathogen-free (SPF) SD rats were selected as research subjects. The, the AD model was created by injecting Aβ1-42 into hippocampus CA1 region of AD rats using a microscopic syringe. Besides, Morris water maze test and Western blot were performed to detect the cognitive function, the levels of destination protein and active oxidation products in the brain of rats. Compared to the Sham group, the escape latency and the distance of the model group significantly increased (*P < *0.05), and the number of times to pass the target quadrant was significantly reduced (*P < *0.05); the expression levels of E2F-1 and NF-κB protein in the hippocampus and the phosphorylation levels of Tau231, Tau262, Tau396, Tau404 and T216-GSK-3β protein of the model group were significantly increased (*P < *0.05); the ROS/RNS value in the hippocampus of the model group significantly increased (*P < *0.05). AD model rats exhibit obvious cognitive dysfunction, which is associated with the activation of E2F-1/NF-κB/GSK-3β signaling pathway and the heightened Tau protein phosphorylation level.

## 1. Introduction

Alzheimer’s disease (AD) is a neurodegenerative disease with a complicated pathology. It is more common in the elderly over 65 years old [1,2]. At present, AD has become an important cause that seriously endangers human health worldwide, second only to heart disease, cancer, and stroke [3]. In China, with the faster population aging, the incidence of AD has shown an increasing trend year by year and the number of patients with AD in China accounts for about 25% of the total number in the world [4]. Clinically, AD manifests as retrograde forgetfulness and changes in personality and emotions [5]. Moreover, the onset of AD is insidious and when it is discovered, it is already very serious and difficult to cure. At the same time, the rehabilitation and care of AD patients are complicated, which brings huge economic and psychological burdens to the patients’ families and society. To find a suitable and efficient treatment method of AD, understanding its pathogenic mechanism is necessary.

The main pathological change of AD is the hyperphosphorylation of Tau protein in the brain nerve cells, which leads to the tangles of nerve fibers, the deposition of extracellular amyloid (Aβ), the formation of senile plaques, and long-term inflammation and neuronal damage [6,7]. The current hypotheses of the pathogenesis of AD include the accumulation of toxic Aβ, abnormal modification of Tau, and gene mutation [8]. GSK-3β is a rate-limiting enzyme of glycogen synthase, which can inhibit the synthesis of liver glycogen [9]. Studies have shown that GSK-3β can modify the function of these proteins by phosphorylation of specific amino acid hydroxyl groups in its downstream substrate protein, and ultimately participate in cell apoptosis and the occurrence of neurodegenerative diseases [10,11]. Nuclear transcription factor κB (NF-κB) is an important multifunctional transcription factor in microglia, which is involved in microglia activation and can induce brain injury caused by inflammation. Studies have shown that over-activated NF-κB can lead to neurological impairment and NF-κB is abnormally expressed in the lesion sites of neurodegenerative diseases [12]. Adenovirus E2 transcription binding factor-1 (E2F1) is also an important transcription factor, which can activate the expression of some genes and promote the change of cell cycle [13]. However, the underlying mechanism of AD remains unclear.

To explore the mechanism of e2F-1 /NF-κB/GSK-3β signaling pathway in the occurrence and development of AD disease, the study built the AD rat model and the relationship between Tau protein phosphorylation and AD disease was analyzed through the E2F-1/NF-κB/GSK-3β pathway. The objective of this study was to understand the pathological mechanism of AD, expected to provide a theoretical basis for the treatment of AD.

## 2. Materials and methods

### 2.1. Research subjects

60 4-month-old SPF-grade Sprague-Dawley (SD) rats were selected as the research subjects, weighed between 250–300 g. They were purchased from Nanjing Junke Bioengineering Co., LTD, and randomly divided into the control group (sham operation group) and AD model group, with 30 rats in each group. All subjects were bred in the SPF animal laboratory of the Experimental Animal Center of XX Hospital and they can move around freely and took a standard diet. The breeding process required a constant temperature of 23.5 ± 1.5°C, a constant humidity of 50 ± 10%, and a 12 h day-night circle. The experiment was carried out under the guidance of *Chinese Laboratory Animal Management Regulations*, and the study was approved by the Animal Care and Use Committee of the Hospital.

### 2.2. Preparation of the AD model

The rats were anesthetized by intraperitoneal injection of 40 mg/kg 1% pentobarbital sodium (Shanghai Civic Chemical Technology Co., LTD., China). The rat’s head was fixed in a stereotaxic device and a 1 cm sagittal incision was made between the eyes and the midline of the ears. Next, an aperture of 0.8 mm was set at 0.8 mm behind the anterior halogen portal and 1.5 mm to the left of the median sagittal line according to the *Stereotaxic Map of Rat Brain*. The AD rat model was prepared by referring to the study of Du et al. (2020) [14]. Subsequently, 4 μL Aβ1-42 (Specification: 2.5 μg/μL, Hangzhou Zhongpeptide Biochemical Co., LTD., China) was slowly injected into the hippocampus CA1 area of the rat using a micro-syringe and the needle was withdrawn 5 min after the injection. Then, the surgical area was smeared with penicillin for disinfection and the skin was disinfected again after sutured. In the control group, the hippocampus CA1 area of the rat was injected with the same amount of normal saline. After the operation, all the rats were kept in separate cages in a warm environment before awakening.

### 2.3. Morris water maze test

The Morris water maze test [15] was performed 7 days after the modeling. The main body of the Morris water maze consists of a stainless-steel circular pool with a height of 100 cm and a diameter of 150 cm. The water temperature is controlled at 23 ± 2°C. A cylindrical escape platform with a diameter of 15 cm and a distance of 2 cm from the water surface is fixed as the first quadrant.

1d prior to the positioning cruise test, the rats were placed on the platform for 30 s and then entered the water to swim freely for 1 min. Next, they were taken out and placed on the platform again to continue learning for 30 s. When the positioning cruise test continued to the 7th day, the rat was placed in other quadrants into the water. If the platform can be found within 90 s and they can stay on the platform for 2 s, the escape latency and distance of the rat were recorded. If the platform cannot be found successfully within 90s, the escape latency was recorded as 90 s.

After the positioning cruise test, the escape platform was taken out and the rat was put into the water from the diagonal quadrant of the target quadrant. Then, the time, path, and the number of crossings of the rat staying in the target quadrant and the diagonal quadrant were recorded.

### 2.4. Western blot

The rat was anesthetized and the hippocampus was quickly separated on ice. After the impurities were removed, the hippocampus was weighed. Next, it was homogenized and the total protein in the tissue was extracted. The BCA kit (Shanghai Biyuntian Biotechnology Co., LTD., China) was used to determine the protein concentration. Subsequently, 10% separation gel and 4% concentration gel were configured, followed by pre-electrophoresis with a constant current of 10 mA. Then, the electrophoresis was started. The target protein bands were transferred from SDS-PAGE glue to PVDF membrane and sealed with 5% skim milk (Shanghai Biyuntian Biotechnology Co., LTD., China) solution for 1 h. Next, diluted E2F-1, NF-κB, GSK-3β, and Tau (Shanghai Aibokang Trading Co., LTD., China) were added, followed by incubation overnight at 4°C. After washing the membrane, the second antibody with horseradish peroxidase conjugation was added (Shanghai Aibokong Trading Co., LTD., China), followed by incubation at room temperature for 1 h. After the membrane was washed, the target protein bands were scanned by gel imager, and the gray value of the target protein bands was analyzed by ImageJ software. β-actin was used as an internal reference and the relative expression of the target protein was detected.

### 2.5. ROS/RNS detection of tissue active oxidation products

The ROS/RNS content of active oxidation products in tissue was detected according to the instructions of frozen section ROS/RNS fluorescence detection Kit (Shanghai Boming Biological Company, China). The freeze-preserved reagent B was mixed with the diluent in a ratio of 1:100 to prepare dyeing solution, followed by pre-cooling on ice. Then, the frozen tissue sections were taken out and washed at room temperature. Next, the tissue was covered with the staining solution and incubated for 20 min at 37°C under dark conditions. Again, the tissue was washed. subsequently, glycerol was used to seal the section. Under the fluorescence microscope, the excitation wavelength of 480 nm~500 nm was set for color development, and the average fluorescence intensity in the image was calculated by using ImageJ software.

### 2.6. Statistical processing

All experimental data were expressed as mean±standard deviation, and SPSS19.0 software was used for statistical analysis. The independent sample *t*-test was used to compare the differences between the groups and *P < *0.05 was considered to be statistically significant.

## 3. Results

To explore the specific biological mechanism of the occurrence and development of AD, and to understand the correlation between the activation of E2F-1/NF-κB/GSK-3β pathway and the cognitive dysfunction of AD, the study prepared the AD rat model. Morris water Maze test was used to investigate the changes of cognitive function in AD rats. Western blot and ROS/RNS kit were used to detect the changes of E2F-1/NF-κB/GSK-3β pathway and oxidative stress during the development and progression of AD.

### 3.1. Positioning cruise test of Morris water maze

**Figure 1.**
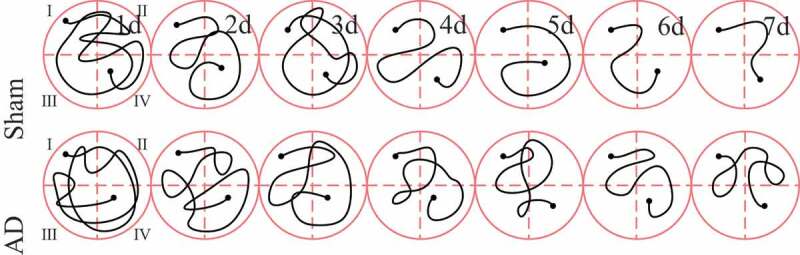
The positioning and cruising path of the rats in Morris’s water maze

**Figure 2.**
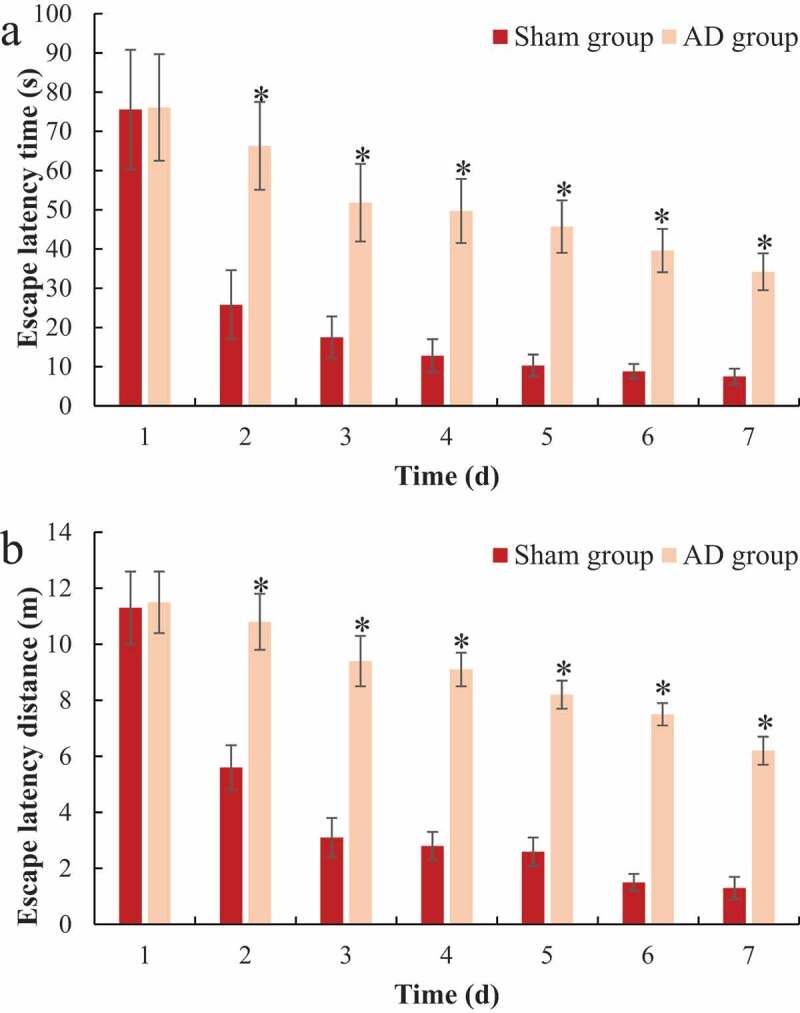
Comparison of the positioning and cruising ability of the rats in Morris water maze

The Morris water maze tested the positioning and cruising ability of each group of rats during the training process and the results were shown in Figures 1 and 2. It was noted from Figure 1 that with the passage of training time, the positioning cruising path of rats in the sham operation group was significantly shortened. Then, the two groups were compared for the escape latency time and distance. As shown in Figure 2(a, b), there was no significant difference between the escape latency time and distance of the two groups of rats on the first day of training (*P > *0.05); from the second to the seventh day of training, the escape latency time and distance in the AD group were significantly longer than those in the sham operation group, and the difference was statistically significant (*P < *0.05).

A was the difference of the escape latency time of rats; B was the difference of the escape latency distance of rats; *P < 0.05.

### 3.2. Space exploration experiment in Morris water maze

**Figure 3.**
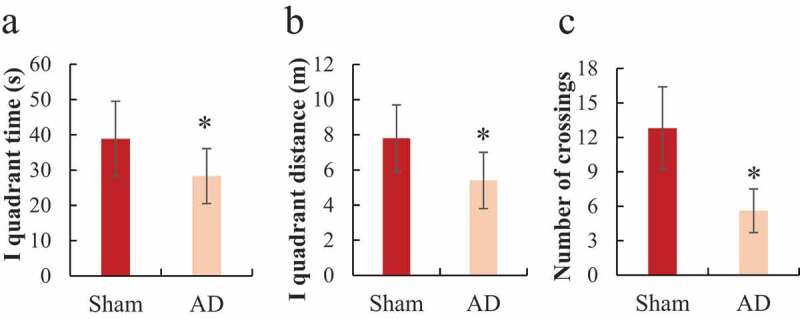
Comparison of space exploration ability of the rats in Morris water maze

The Morris water maze tested the space exploration ability of each group of rats during the training process and the results were shown in Figure 3. It was noted that the sham operation group had longer residence time in the first quadrant, a longer total path in the first quadrant, and more times crossing the target quadrant than the AD group, and the difference was statistically significant (*P < *0.05).

(a) was the difference in the residence time of the rats in the first quadrant; (b) was the difference in the total path of the rats in the first quadrant; (c) was the difference in the times the rats crossed the first quadrant; *P < 0.05.

### 3.3. Western blot to detect E2F-1, NF-κB, and GSK-3β protein levels in the hippocampus of rats

**Figure 4.**
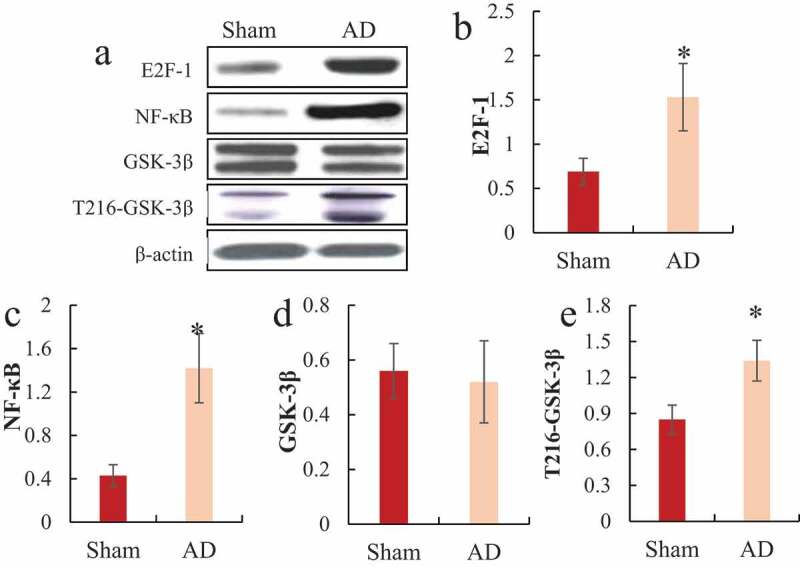
Comparison of E2F-1, NF-κB, GSK-3β, and T216-GSK-3β protein expression in the hippocampus of rats

Western blot detected the differences in the protein expression levels of E2F-1, NF-κB, GSK-3β, and T216-GSK-3β in the hippocampus of the two groups of rats, and the results were shown in Figure 4. It was noted that the protein expression levels of E2F-1, NF-κB, and T216-GSK-3β in the hippocampus of the AD model group were significantly higher than those of the sham operation group, and the difference was statistically significant (*P < *0.05); while there was no significant difference in the expression level of GSK-3β protein in the hippocampus of rats between the two groups (*P > *0.05).

A was the Western blot strip diagram; B was the relative expression level of E2F-1; C was the relative expression level of NF-κB; D was the relative expression level of GSK-3β; E was the relative expression level of T216-GSK-3β; *P < 0.05

### 3.4. Western blot to detect the phosphorylation level of Tau protein in the hippocampus of rats

**Figure 5.**
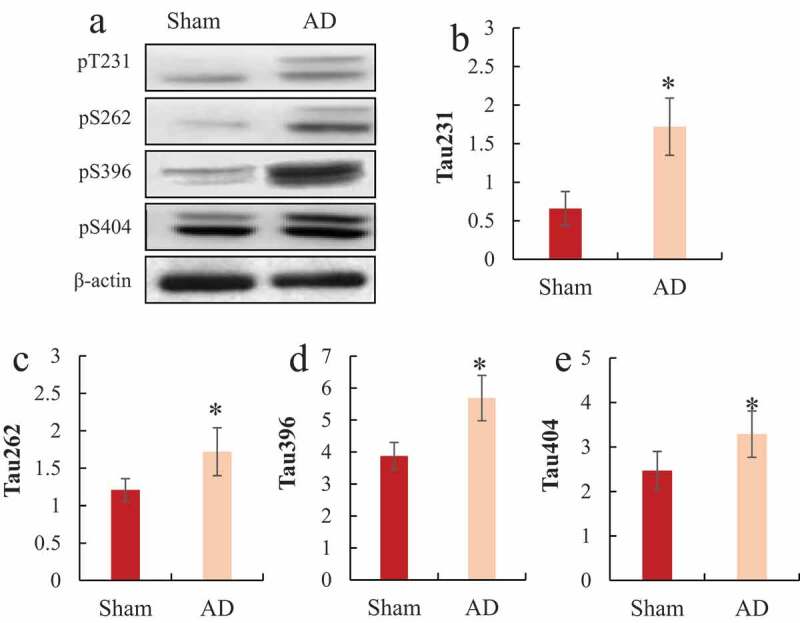
Comparison of Tua protein expression levels in the hippocampus of rats

Western blot detected the differences in the phosphorylation levels of Tau proteins pT231, pS262, pS396, and pS404 in the hippocampus of the two groups of rats, and the results were shown in Figure 5. It was noted that the protein phosphorylation levels of Tau pT231, pS262, pS396, and pS404 in the hippocampus of the AD model group were significantly higher than those of the sham operation group, and the difference was statistically significant (*P < *0.05).

A was the Western blot strip chart; B was the relative expression level of Tua pT231; C was the relative expression level of Tua pS262; D was the relative expression level of Tua pS396; E was the relative expression level of Tua pS404; *P < 0.05.

### 3.5. Immunofluorescence staining to analyze the changes of reactive oxygen species (ROS/RNS) in the hippocampus of rats

**Figure 6.**
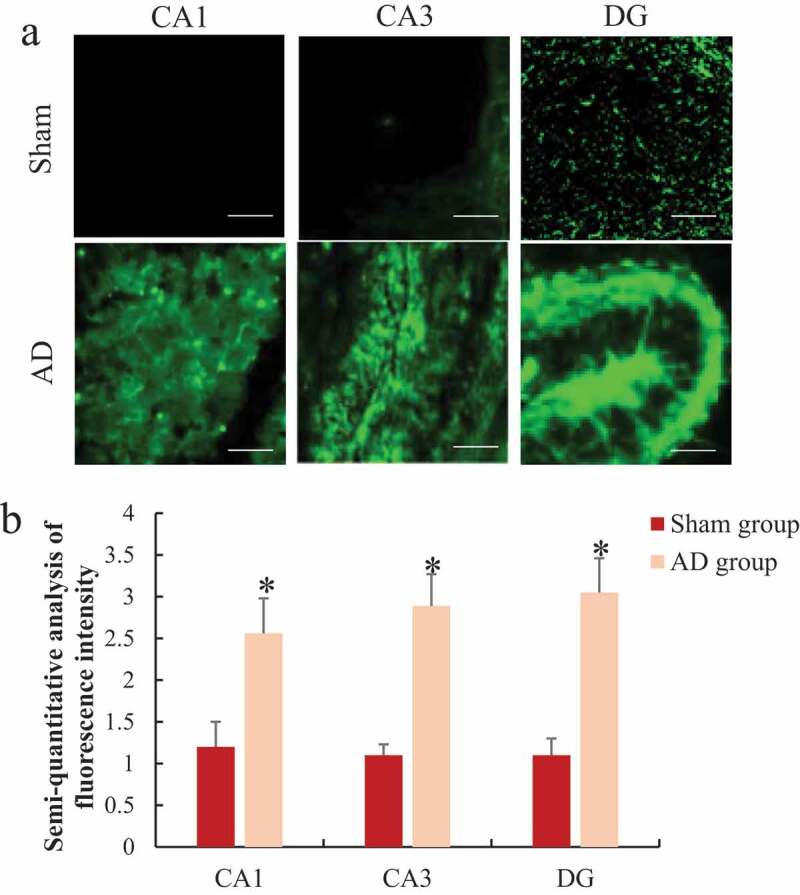
Comparison of ROS/RNS differences in the hippocampus group of rats

The differences of ROS/RNS in CA1, CA3, and DG in the hippocampus of rats were analyzed by immunofluorescence staining and the results are shown in Figure 6. It was noted that the level of ROS/RNS in the hippocampus CA1, CA3, and DG of the AD group were 2.13 times, 2.63 times, and 2.77 times that of the sham operation group, respectively, significantly higher than the sham operation group, the difference was statistically significant (*P < *0.05).

A was the immunofluorescence staining of each part of the hippocampus (Bar = 1 μm); B was the semi-quantitative result of ROS/RNS fluorescence intensity; *P < 0.05.

## 4. Discussion

As a neurodegenerative disease, AD occurs mostly in the elderly. It clinically manifests as retrograde forgetting, cognitive dysfunction, and personality changes [16,17]. The current hypotheses about the onset of AD mainly include the effect of toxic Aβ, abnormality of Tau protein, long-term inflammation, and gene mutations [7,18,19]. Abnormal synthesis of Aβ or the body’s dysfunction to remove Aβ can both cause the deposition or accumulation of Aβ in the brain, which in turn leads to AD [20]. In the process of amyloidosis, Aβ will be cleaved by γ and β secretase into Aβ40 and Aβ42, of which Aβ42 has strong neuronal toxicity [21]. At present, the AD model is constructed by the intraventricular injection of Aβ and compared with the AD model obtained by mutating Tau gene, it is economical and more practical, and demonstrates better effects [22,23]. Therefore, in the study, the AD model of rats was constructed by injecting Aβ1-42 into the hippocampus. Studies have shown that intracerebroventricular injection of Aβ amyloid fragments can increase the deposition of Aβ in the brain, causing neuronal damage and reduction of nerve synapses, and the learning and memory ability of model animals will also decrease [24,25]. In this study, the positioning cruise experiment and space exploration test in the Morris water maze were carried out to analyze the changes in the positioning cruise ability and space exploration ability of rats after intraventricular injection of Aβ1-42. The results found that AD rats’ escape latency time was significantly prolonged, and the space exploration ability significantly decreased. It suggested that the learning ability and spatial memory of rats were significantly reduced after intraventricular injection of Aβ1-42 and the AD model was successfully constructed, which laid the foundation for subsequent research.

The phosphorylation of Tau protein in some special sites is associated with the occurrence and development of AD. Studies have confirmed that there are obvious phosphorylation modifications of Thr181, Thr231, Ser235, Tyr394, Ser396, and Ser404 in the Tau protein in the brain of AD patients, which suggested that the protein was closely associated with neurodegeneration [26]. Tau phosphorylation at Thr231 was the first factor that was proved to be related to the progression of AD disease [27]. The phosphorylation of Ser262 site can accelerate the accumulation of Tau protein, and the hyperphosphorylation modification of Tau protein can reduce the memory preservation ability, and even lead to memory loss [28]. Other studies have confirmed that Ser404 site can modify Tau protein [29]. In the study, Tau protein phosphorylation of Thr231, Ser262, Ser396, and Ser404 sites was detected to assess the condition of AD rats.

The Ser404 site in Tau protein is a specific site in AD induced by GSK-3β activation [30]. GSK-3β is the earliest discovered Tau protein-related kinase, which participates in Tau protein-related pathological changes and amyloid pathological changes. Studies have shown that GSK-3β is related to the apoptosis of nerve cells, the atrophy of nerve protrusions, and cognitive impairment [31,32]. The results of this study showed that there was no significant difference in the total amount of GSK-3β protein between AD rats and normal rats, but the expression level of T216-GSK-3β protein in AD model rats was significantly decreased. At the same time, the results showed that compared with normal rats, the Tau protein phosphorylation levels at Thr231, Ser262, Ser396, and Ser404 sites in AD rats were significantly increased, which further confirmed the Tau protein phosphorylation levels at these sites and GSK-3β were related to the occurrence of AD. Therefore, it is important to find drugs that can control GSK-3β or Tau protein hyperphosphorylation for the treatment of AD.

Neurodegenerative diseases are caused by many factors, such as oxidative stress, inflammatory response, neuronal apoptosis, and autophagy dysfunction. Oxidative stress and neuroinflammatory response can accelerate the process of neurodegeneration, while oxidation and antioxidant effects under pathological conditions can cause the accumulation of reactive oxygen species and free radicals, and the generation of a large amount of reactive oxygen can cause oxidative damage reaction [33]. In living tissue or cells, if oxygen free radical generation or clearance is inhibited, and excessive accumulation of ROS/RNS in cells will destroy the structure of cell membranes and interfere with energy metabolism inside cells, resulting in cell dysfunction or apoptosis [34]. As AD progresses, oxidative stress is closely related to the pathological process of Aβ and Tau. The increase in the phosphorylation of Tau in the cell and the excessive accumulation of Aβ will aggravate the oxidative stress damage of the cell [35]. The results of this study showed that ROS/RNS levels in hippocampal CA1, CA3, and DG were significantly increased in AD rats, and that microglia can be activated during oxidative stress and mediate neurodegeneration and injury. With the activation of microglia, it can promote the secretion of pro-inflammatory factors, and then cause inflammation. NF-κB is an important target of anti-inflammation and antioxidant stress [36]. E2F1 is an important transcription factor regulating cell cycle, and overexpression of E2F1 can induce neuronal apoptosis [37]. The results of this study showed that NF-κB and E2F1 expression levels were also significantly increased, indicating that there existed significant oxidative stress, neuroinflammatory response, and apoptosis of nerve cells in AD.

## 5. Conclusion

In the study, the AD rat model was constructed by intraventricular injection of Aβ to explore the pathogenic mechanism of AD. The results showed that the learning ability and spatial memory ability of the AD rat model were significantly reduced, and the expression levels of E2F-1, NF-κB, and GSK-3β protein in the brain tissue had obvious changes. At the same time, Tau protein phosphorylation levels at Thr231, Ser262, Ser396, and Ser404 sites in the hippocampus of AD rats were significantly increased, and significant oxidative stress damage occurred. The results of this study were intended to understand the pathogenesis of AD and provide a theoretical basis for the early diagnosis and targeted therapy of the disease. However, this study only explores the pathogenesis of AD and does not explore the mechanism of action of targeted therapy drugs for the treatment of AD. In the follow-up, further efforts are needed to find potential therapeutic drugs for the treatment of AD animal models and to explore the regulatory mechanism of the drug on the E2F-1/NF-κB/GSK-3β pathway and Tau protein phosphorylation. In conclusion, the study provides a reference for improving the clinical efficacy of AD.
